# Cutaneous infection due to *Bacillus cereus*: a case report

**DOI:** 10.1186/s12879-022-07372-9

**Published:** 2022-04-21

**Authors:** Mohammad Esmkhani, Saeed Shams

**Affiliations:** 1 Ali Ebne Abitaleb Hospital, Qom, Iran; 2 Cellular and Molecular Research Center, Qom University of Medical Sciences, Qom, Iran

**Keywords:** *Bacillus cereus*, Cutaneous infection, 16S rRNA, Sequencing

## Abstract

**Background:**

*Bacillus cereus* is a Gram-positive bacterium that can be found in various natural and human-made environments. It is often involved in gastrointestinal infections and food poisoning; yet, it can rarely cause serious non-gastrointestinal tract infections.

**Case presentation:**

Here we describe a case of *B. cereus* cutaneous infection of a wound on the hand of a young woman from a rural area in Iran. On admission, she had no systemic symptoms other than a cutaneous lesion. The identification of the causative agent was performed using sequencing of the 16S rRNA gene of the bacteria isolated from the wound. The isolated microorganism was identified as *B. cereus*. Targeted antibiotic therapy with ciprofloxacin was successful.

**Discussion and conclusion:**

Although non-intestinal infections caused by *B. cereus* are rare, it should be taken into consideration that this organism might also cause infections in other parts of the body.

## Background

*Bacillus* (*B.*) *cereus* is a rod-shaped, Gram-positive, endospore-forming aerobic or facultative anaerobic, motile bacterium widely distributed in natural environments [1, 2]. Although *B. cereus* is considered as an opportunistic bacterium, some strains can cause food poisoning which is associated with the synthesis of an emetic toxin e.g. in cooked foods, such as rice, wet and dry wheat noodles, spices, grains, legumes, and legume products [3]. It can also cause gastrointestinal infections linked to the bacterium growth in the host intestines and production of a set of enterotoxins [4, 5]. Although *B. cereus* non-gastrointestinal infections are rare, they can also cause bacteremia, meningitis, endocarditis, endophthalmitis, pneumonia, and soft tissue infections [6–8]. Skin infections due to *B. cereus* have been sporadically described in the literature. Except for a case report in an immunocompetent patient [9], the rest of the primary cutaneous infection cases caused by this bacterium have been associated with underlying conditions such as diabetes, traumatic injuries, immunocompromised states, etc. [10–14]. Gastrointestinal and non-gastrointestinal pathogenicity of *B. cereus* is associated with the secretion of various toxins, including tissue-destructive exoenzymes [6, 15, 16]. Here, we present a patient with no underlying disease and cutaneous ulcer caused by *B. cereus*.

## Case presentation

**Fig. 1:**
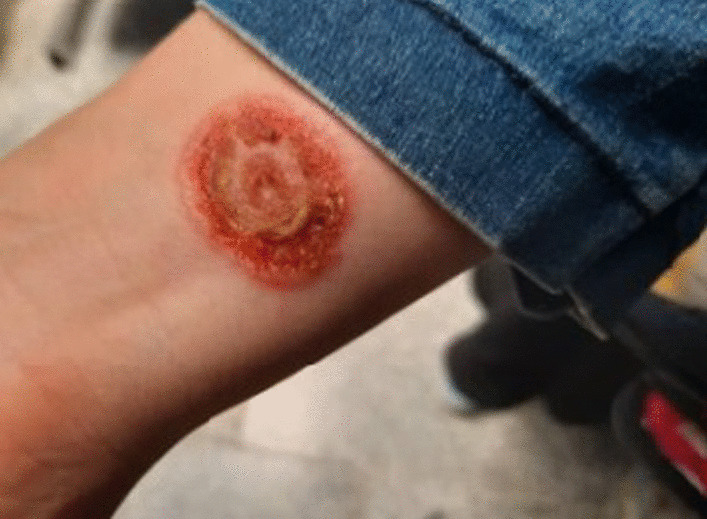
Patient wound at the time of referral and sampling

A 33-year-old woman, living in a rural area in the vicinity of Qom, Iran, without any underlying disease visited our center with the chief complaint of a wound on her right wrist. According to the patient, the wound had developed following small vesicles, while she had no history of trauma such as cut, scratch, scrape, puncture wound, bruise, etc. The wound was painless with no discharge, and the patient had decided to have a medical examination 10 days after the onset of the symptoms due to the progression and appearance of the wound and its itching (Fig. 1). Following physical examination, no systemic symptoms were observed in the patient.

## Laboratory findings

### Sampling and isolation of the infectious agent

**Fig. 2:**
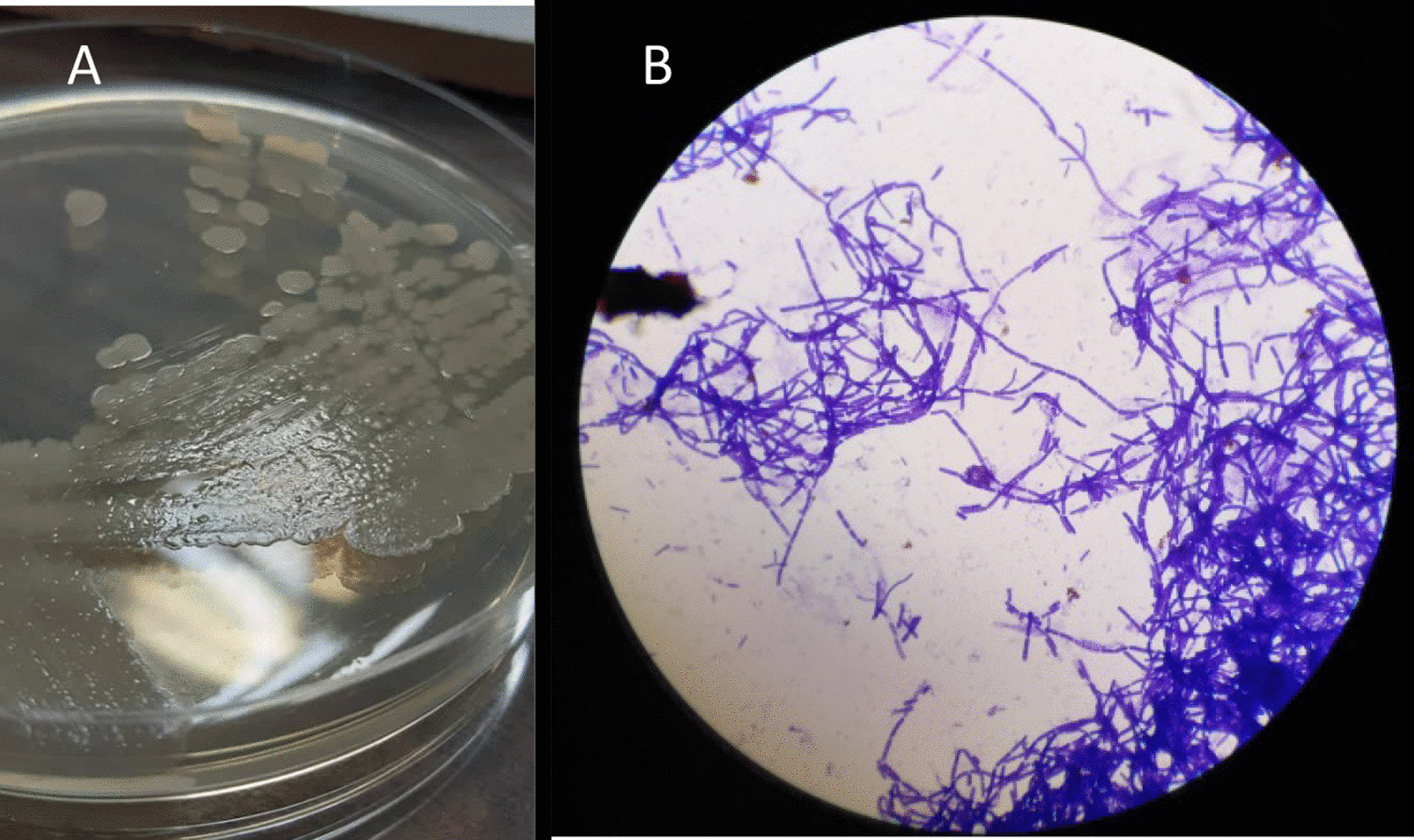
**A** Colonies of *B. cereus* subcultured on nutrient agar; **B** microscopic image at × 1000 magnification

Sampling was performed according to the study of Parikh et al. [17]. Briefly, the infected area was disinfected using 70% isopropyl alcohol and allowed to dry for 60 s. For sample collection, aspiration was carried out using a needle (fine-needle aspiration) from the edge and under of the infection site. The sample was cultured on blood agar, chocolate agar, and eosin methylene blue agar (Merck-Germany), and was incubated for 24 h at 37 °C. After incubation, the bacteria grew well on blood agar and chocolate agar, and pure colonies with non-pigmented, dry, round, flat or slightly convex, opaque, milky, and irregular edges appearance were observed. In Gram staining, chains of large Gram-positive bacilli were detected (Fig. 2).

### Molecular evaluation for detecting the target bacterium

DNA extraction from isolated colonies was performed using FavorPrep™ Tissue Genomic DNA Extraction Mini Kit (Favorgen Biotech, Ping-Tung, Taiwan) according to the manufacturer’s instructions. PCR was performed on the 16S rRNA gene using universal 27F (5′-AGAGTTTGATCCTGGCTCAG-3′) and 1492R (5′-TACGGYTACCTTGTTACGACTT-3′) with initial denaturation conditions (96 °C, 4 min) and in 30 cycles, including denaturation (94 °C, 30 s), annealing (57 °C, 30 s), extension (72 °C, 1 min) and final extension (72 °C, 10 min) [18] in a thermocycler (Eppendorf, Hamburg, Germany). The PCR product was examined on 1% agarose gel and then sent to Codon Genetic Group, Tehran, Iran, for sequencing. BLAST was performed at the NCBI website (https://blast.ncbi.nlm.nih.gov/Blast.cgi), and the result revealed that the isolated bacterium was *Bacillus cereus*.

### Antibiotic susceptibility testing (AST) and treatment

**Fig. 3:**
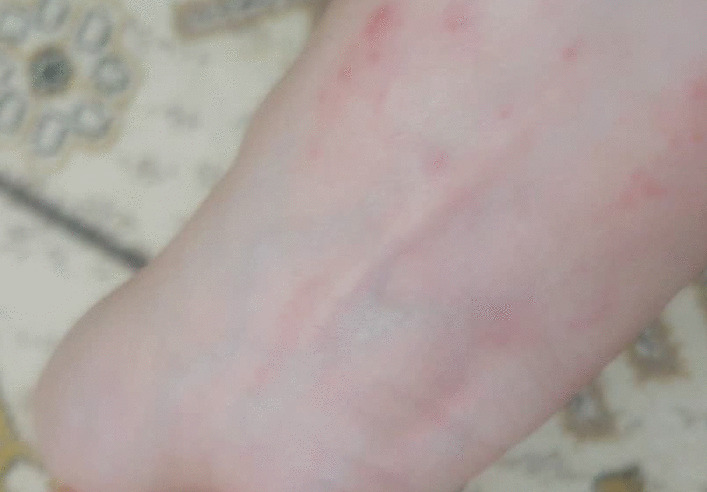
Wound appearance after antibiotic therapy

AST of the target *B. cereus* was performed according to Clinical & Laboratory Standards Institute (CLSI) instructions [19]. The results showed that the strain was susceptible to gentamicin, ciprofloxacin, chloramphenicol, and tetracycline. Following consultation with two infectious disease specialists, treatment was performed with oral administration of ciprofloxacin (500 mg twice a day for 14 days). During treatment, the patient's ulcer commenced to heal. Figure 3 shows the patient's cutaneous infection after 18 days of antibiotic therapy.

## Discussion and conclusion

*B. cereus*, a soil-dwelling organism, is present in various environments and is mostly isolated from food sources [3]. This species is primarily responsible for two types of gastrointestinal illnesses, e.g. food poisoning associated with vomiting and intestinal infections manifested by diarrhea. There are numerous reports of other severe infections such as bacteremia, meningitis, etc. caused by *B. cereus*, mainly reported in immunocompromised patients, injecting drug users, neurosurgical patients with intraventricular shunts, patients with neoplastic processes, trauma, or nosocomial infections [6–8, 15, 20]. In addition, few reports of skin infections caused by this microorganism have been published. Michelotti et al. informed about *B. cereus* causing a necrotic cutaneous infection in a patient with type 2 diabetes mellitus [10], while Sánchez-Hernández et al. reported a cutaneous infection due to *B. cereus* in a 13-year-old child who ultimately required surgical treatment and skin grafting [21]. Another report by Pillai, who emphasized on *B. cereus* as a forgotten agent, showed a case of surgical site infection caused by this bacterium after fasciotomy in a 31-year-old man hospitalized in the orthopedic ward with a comminuted fracture of the tibia [22]. Cutaneous involvement with *B. cereus* infections has also been reported in immunocompromised patients with neutropenia [11], granulocytopenia [23], etc.

This bacterium produces several virulence factors, including hemolysin BL (HBL), non-hemolytic enterotoxin (Nhe) or cytotoxin K (CytK), etc., which have hemolytic, cytotoxic, dermonecrotic, and vascular permeability activities in the animal models [15, 16, 24]. In fact, a wide range of effects on the skin can be seen, ranging from superficial necrosis to necrotizing fasciitis and myonecrosis [10].

In this study, we isolated *B. cereus* from a cutaneous lesion on the hand. Most infectious disease specialists, dermatologists, and even clinical microbiologists seem to pay less attention to this bacterium as a causative agent of skin infections. Compared to other similar papers, the image of the wound published in this report was of a completely different appearance and the first from an immunocompetence patient with no underlying disease. Dermatological manifestations of this organism however could be confusing, mimicking many other skin infections. Therefore, microbial culture can be helpful for diagnosis. In addition, the patient had no history of trauma. It seems that in non-traumatic cases, the entry of the bacteria can be through microscopic scratches on the skin of the hands and/or feet [10]. As previously mentioned, our patient was not immunocompromised and had no underlying disease, similar to the first report of cutaneous *B. cereus* infection in an immunocompetent patient published by Boulinguez et al. in 2002 [9].

The bacterium isolated from our patient was susceptible to some tested antibiotics, and treatment of the patient with ciprofloxacin was consequently effective. Antibiotic resistance of this bacterium species has also been reported in some studies. In a study conducted by Ikeda et al., 48.3–100%, 65.5%, and 10.3% of *B. cereus* strains isolated from bloodstream infection were resistant to cephalosporins, clindamycin, and levofloxacin, respectively, while all of them were susceptible to vancomycin, gentamicin, and imipenem [25].

Overall, this study is one of the first to demonstrate cutaneous infection due to *B. cereus*, especially in the immunocompetence. Our report emphasizes on the importance of vigilance against a rare but potentially serious non-gastrointestinal infection caused by this bacterium. Therefore, *B. cereus* isolated from any part of the body, especially traumatic, non-traumatic, and surgical wounds, should not be considered as an environmental contaminant without accurate evaluation.

### Limitations

Due to some financial constraints, we were unable to evaluate the virulence factors of the target bacterium (especially toxins produced).

## Data Availability

The datasets used and/or analyzed during the current study are available from the corresponding author on reasonable request.

## Abbreviations

16S rRNA: 16S ribosomal ribonucleic acid
DNA: Deoxyribonucleic acid
PCR: Polymerase chain reaction
NCBI: National Center for Biotechnology Information
BLAST: Basic Local Alignment Search Tool
AST: Antibiotic susceptibility testing
CLSI: Clinical & Laboratory Standards Institute
HBL: Hemolysin BL
CytK: Cytotoxin K
Nhe: Non-hemolytic enterotoxin
